# 25,26,27,28-Tetra­butoxy-5,11,17,23-tetra-*tert*-butyl­calix[4]arene chloro­form tetra­solvate dihydrate

**DOI:** 10.1107/S160053680902217X

**Published:** 2009-06-27

**Authors:** Zhengyi Li, Dinghao Yuan, Haitao Xi, Xiaoqiang Sun

**Affiliations:** a Key Laboratory of Fine Chemical Engineering, Jiangsu Polytechnic University, Changzhou 213164, Jiangsu, People’s Republic of China; b Gaochun County Environmental Protection Bureau, No. 5, Xuhe North Road, Chunxi Town, Gaochun city 211300, Jiangsu, People’s Republic of China

## Abstract

The title compound, C_60_H_88_O_4_·4CHCl_3_·2H_2_O, is the alkyl­ated product of 5,11,17,23-tetra-*tert*-butyl­calix[4]arene. It adopts a distorted cone conformation which leads to an open cavity. All the phenolic rings are tilted so that their *tert*-butyl groups are pitched away from the calix cavity. Two opposite aromatic rings are close to being perpendicular to one another [dihedral angle 85.0 (2)°], while the other pair of opposite rings is almost parallel [dihedral angle 8.1 (2)°], and adjacent phenolic rings are almost perpendicular [dihedral angles 82.4 (1) or 87.9 (1)°]. In the crystal, the water molecule and calixarene interact by way of O—H⋯O hydrogen bonds.

## Related literature

For calix[4]arene derivatives as supra­molecular building blocks, see: Böhmer (1995 ▶); Homden & Redshaw (2008 ▶). For related structures, see: Rathore *et al.* (2000 ▶) and Brusko *et al.* (2005 ▶). For details of the synthesis, see: Matthews *et al.* (1999 ▶).
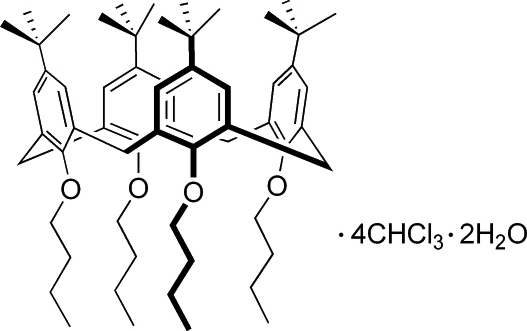


## Experimental

### 

#### Crystal data

C_60_H_88_O_4_·4CHCl_3_·2H_2_O
*M* *_r_* = 1386.81
Orthorhombic, 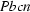

*a* = 23.697 (6) Å
*b* = 13.682 (6) Å
*c* = 25.402 (11) Å
*V* = 8236 (5) Å^3^
*Z* = 4
Mo *K*α radiation
μ = 0.44 mm^−1^
*T* = 291 K
0.26 × 0.22 × 0.20 mm

#### Data collection

Bruker SMART APEX CCD diffractometer
Absorption correction: multi-scan (*SADABS*; Bruker, 2000 ▶) *T*_min_ = 0.894, *T*_max_ = 0.917
30239 measured reflections
8098 independent reflections
5524 reflections with *I* > 2σ(*I*)
*R*_int_ = 0.036

#### Refinement

*R*[*F* ^2^ > 2σ(*F* ^2^)] = 0.053
*wR*(*F* ^2^) = 0.117
*S* = 1.06
8098 reflections
396 parameters
H-atom parameters constrained
Δρ_max_ = 0.82 e Å^−3^
Δρ_min_ = −0.73 e Å^−3^

### 

Data collection: *SMART* (Bruker, 2000 ▶); cell refinement: *SAINT* (Bruker, 2000 ▶); data reduction: *SAINT*; program(s) used to solve structure: *SHELXTL* (Sheldrick, 2008 ▶); program(s) used to refine structure: *SHELXTL*; molecular graphics: *SHELXTL*; software used to prepare material for publication: *SHELXTL*.

## Supplementary Material

Crystal structure: contains datablocks I, global. DOI: 10.1107/S160053680902217X/ez2170sup1.cif

Structure factors: contains datablocks I. DOI: 10.1107/S160053680902217X/ez2170Isup2.hkl

Additional supplementary materials:  crystallographic information; 3D view; checkCIF report

## Figures and Tables

**Table 1:** Hydrogen-bond geometry (Å, °)

*D*—H⋯*A*	*D*—H	H⋯*A*	*D*⋯*A*	*D*—H⋯*A*
O3—H3*C*⋯O4	0.85	1.52	2.149 (8)	127
O5—H5*C*⋯O5^i^	0.85	1.74	2.299 (13)	121

Symmetry code: (i) 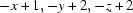.

**Fig. 1.**
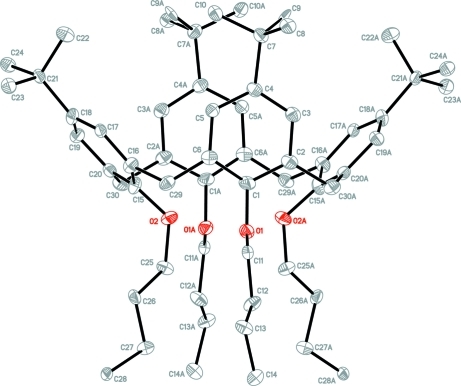
The molecular structure of the title compound. Displacement ellipsoids are drawn at the 25% probability level. H atoms and solvent molecules have been omitted for clarity. [symmetry code: -x + 1, y, -z + 3/2].

## supplementary crystallographic information

### Comment

Derivatives of calix[4]arene, as one of the most important supramolecular building blocks, are useful in ion and metal complexation because they form suitable scaffolds for the development of new bulky and structurally well defined ligands (Böhmer, 1995; Homden & Redshaw 2008). As an important approach to obtain functionalized calixarenes, alkylation of the phenolic hydroxyl groups on the lower rim of the calixarene has been widely explored. The crystal structures of propyl (Rathore *et al.*, 2000) and pentyl (Brusko *et al.*, 2005) alkylated calix[4]arene have been reported. We herein present the structure of the tetrabutyl substituted calix[4]arene (Fig. 1).

The title compound adopts a distorted cone conformation with a small cavity. All phenolic rings are tilted so that their *tert*-butyl groups are pitched away from the calix cavity, as defined by the angles which the aromatic rings make with the plane of the four bridging CH_2_ moieties (C29, C30, C29A and C30A) which link them, *viz.* 94.0 (3)° (C1–C6 or C1A–C6A) and 132.5 (1)° (C15–C20 or C15A–C15A). Two opposite aromatic rings (C15–C20 and C15A–C20A) are close to being perpendicular to one another (dihedral angle 85.0 (2)°) while the other pair of opposite phenolic rings (C1–C6 and C1A–C6A) are almost parallel (dihedral angle 8.1 (2)°), and the adjacent phenolic rings are almost perpendicular (dihedral angles 97.6 (1)° or 92.1 (1)°).

### Experimental

NaH (0.96 g, 40 mmol) and DMF (20 ml) were added to a suspension of 5,11,17,23-tetra(*tert*-butyl)calix[4]arene (3.25 g, 5 mmol) in DMF (30 ml) under argon. The suspension was stirred for 1 h, and then 1-bromobutane (5.48 g, 40 mol) was added. Stirring was continued at room temperature for 2 d. Water (100 ml) was added and the precipitate formed collected by filtration. The solid was dissolved in chloroform and washed with 15% HCl and water. The organic layer was dried and the solvent evaporated. Precipitation from chloroform/methanol gave the title compound as a white solid with sufficient purity (68% yield). Single crystals suitable for X-ray diffraction were obtained by evaporation of an methanol-chloroform solution.

### Refinement

All the H atoms were placed in geometrically idealized positions and constrained to ride on their parent atoms, with C—H distances of 0.93–0.98 Å, and with *U*_iso_(H) = 1.2 or 1.5*U*_eq_(C). H atoms bonded to O atoms were refined independently with isotropic displacement parameters. Each water molecule is located over three sites with refined occupancies of 0.3, 0.3 and 0.4, respectively.

### Crystal data

**Table:**

C_60_H_88_O_4_·4CHCl_3_·2H_2_O	*F*(000) = 2928
*M**_r_* = 1386.81	*D*_x_ = 1.118 Mg m^−^^3^
Orthorhombic, *P**b**c**n*	Mo *K*α radiation, λ = 0.71073 Å
Hall symbol: -P 2n 2ab	Cell parameters from 3284 reflections
*a* = 23.697 (6) Å	θ = 2.1–23.4°
*b* = 13.682 (6) Å	µ = 0.44 mm^−^^1^
*c* = 25.402 (11) Å	*T* = 291 K
*V* = 8236 (5) Å^3^	Block, colourless
*Z* = 4	0.26 × 0.22 × 0.20 mm

### Data collection

**Table:**

Bruker SMART APEX CCD diffractometer	8098 independent reflections
Radiation source: sealed tube	5524 reflections with *I* > 2σ(*I*)
graphite	*R*_int_ = 0.036
φ and ω scans	θ_max_ = 26.0°, θ_min_ = 1.6°
Absorption correction: multi-scan (*SADABS*; Bruker, 2000)	*h* = −29→29
*T*_min_ = 0.894, *T*_max_ = 0.917	*k* = −16→16
30239 measured reflections	*l* = −30→31

### Refinement

**Table:**

Refinement on *F*^2^	Primary atom site location: structure-invariant direct methods
Least-squares matrix: full	Secondary atom site location: difference Fourier map
*R*[*F*^2^ > 2σ(*F*^2^)] = 0.053	Hydrogen site location: inferred from neighbouring sites
*wR*(*F*^2^) = 0.117	H-atom parameters constrained
*S* = 1.06	*w* = 1/[σ^2^(*F*_o_^2^) + (0.05*P*)^2^ + 1.66*P*] where *P* = (*F*_o_^2^ + 2*F*_c_^2^)/3
8098 reflections	(Δ/σ)_max_ < 0.001
396 parameters	Δρ_max_ = 0.82 e Å^−^^3^
0 restraints	Δρ_min_ = −0.73 e Å^−^^3^

### Special details

**Table:**

Geometry. All e.s.d.'s (except the e.s.d. in the dihedral angle between two l.s. planes) are estimated using the full covariance matrix. The cell e.s.d.'s are taken into account individually in the estimation of e.s.d.'s in distances, angles and torsion angles; correlations between e.s.d.'s in cell parameters are only used when they are defined by crystal symmetry. An approximate (isotropic) treatment of cell e.s.d.'s is used for estimating e.s.d.'s involving l.s. planes.
Refinement. Refinement of *F*^2^ against ALL reflections. The weighted *R*-factor *wR* and goodness of fit *S* are based on *F*^2^, conventional *R*-factors *R* are based on *F*, with *F* set to zero for negative *F*^2^. The threshold expression of *F*^2^ > σ(*F*^2^) is used only for calculating *R*-factors(gt) *etc*. and is not relevant to the choice of reflections for refinement. *R*-factors based on *F*^2^ are statistically about twice as large as those based on *F*, and *R*- factors based on ALL data will be even larger.

### Fractional atomic coordinates and isotropic or equivalent isotropic displacement parameters (Å^2^)

**Table:**

	*x*	*y*	*z*	*U*_iso_*/*U*_eq_	Occ. (<1)
C1	0.57787 (15)	0.8058 (2)	0.67567 (14)	0.0420 (8)	
C2	0.54138 (13)	0.8518 (2)	0.63953 (12)	0.0404 (7)	
C3	0.54206 (12)	0.9534 (2)	0.63508 (11)	0.0386 (6)	
H3	0.5179	0.9848	0.6117	0.046*	
C4	0.57917 (13)	1.0070 (2)	0.66598 (11)	0.0421 (7)	
C5	0.61583 (11)	0.9617 (2)	0.70150 (10)	0.0332 (6)	
H5	0.6406	0.9986	0.7218	0.040*	
C6	0.61457 (12)	0.8602 (2)	0.70603 (12)	0.0370 (7)	
C7	0.57813 (12)	1.1231 (2)	0.65850 (11)	0.0387 (6)	
C8	0.60471 (12)	1.1455 (2)	0.60597 (11)	0.0393 (7)	
H8A	0.5857	1.1999	0.5901	0.059*	
H8B	0.6016	1.0894	0.5834	0.059*	
H8C	0.6438	1.1613	0.6109	0.059*	
C9	0.51820 (10)	1.1628 (2)	0.66195 (11)	0.0357 (6)	
H9A	0.5188	1.2325	0.6574	0.054*	
H9B	0.5025	1.1472	0.6958	0.054*	
H9C	0.4955	1.1337	0.6348	0.054*	
C10	0.61488 (11)	1.1702 (2)	0.69931 (12)	0.0390 (7)	
H10B	0.6530	1.1475	0.6953	0.058*	
H10C	0.6014	1.1534	0.7338	0.058*	
H10A	0.6138	1.2399	0.6950	0.058*	
C11	0.61946 (12)	0.6586 (2)	0.64961 (11)	0.0367 (6)	
H11A	0.6549	0.6739	0.6668	0.044*	
H11B	0.6208	0.6851	0.6142	0.044*	
C12	0.61228 (12)	0.5483 (2)	0.64683 (12)	0.0401 (7)	
H12A	0.6035	0.5234	0.6816	0.048*	
H12B	0.5809	0.5328	0.6238	0.048*	
C13	0.66489 (12)	0.4991 (2)	0.62667 (11)	0.0372 (6)	
H13A	0.6957	0.5062	0.6516	0.045*	
H13B	0.6763	0.5266	0.5931	0.045*	
C14	0.64812 (11)	0.3916 (2)	0.62061 (11)	0.0371 (6)	
H14B	0.6554	0.3576	0.6529	0.056*	
H14C	0.6698	0.3626	0.5928	0.056*	
H14A	0.6087	0.3874	0.6123	0.056*	
C15	0.58547 (13)	0.7960 (3)	0.82537 (12)	0.0386 (7)	
C16	0.63350 (12)	0.8410 (2)	0.80232 (12)	0.0381 (7)	
C17	0.65826 (12)	0.9148 (2)	0.82949 (10)	0.0357 (6)	
H17	0.6889	0.9475	0.8148	0.043*	
C18	0.63800 (12)	0.9434 (2)	0.88036 (12)	0.0425 (7)	
C19	0.59227 (12)	0.9009 (2)	0.90043 (12)	0.0382 (6)	
H19	0.5790	0.9204	0.9332	0.046*	
C20	0.56358 (12)	0.8264 (2)	0.87252 (11)	0.0344 (6)	
C21	0.66801 (11)	1.0294 (2)	0.90845 (10)	0.0350 (6)	
C22	0.66772 (13)	1.1203 (2)	0.87343 (12)	0.0440 (7)	
H22A	0.6870	1.1726	0.8910	0.066*	
H22B	0.6295	1.1393	0.8664	0.066*	
H22C	0.6865	1.1060	0.8409	0.066*	
C23	0.73076 (11)	1.0099 (2)	0.91302 (11)	0.0391 (6)	
H23A	0.7512	1.0576	0.8929	0.059*	
H23B	0.7390	0.9457	0.8998	0.059*	
H23C	0.7419	1.0139	0.9493	0.059*	
C24	0.64602 (12)	1.0517 (2)	0.96417 (11)	0.0387 (6)	
H24A	0.6342	0.9921	0.9807	0.058*	
H24B	0.6146	1.0957	0.9620	0.058*	
H24C	0.6755	1.0813	0.9846	0.058*	
C25	0.55579 (12)	0.6195 (2)	0.80020 (11)	0.0360 (6)	
H25A	0.5388	0.5939	0.7683	0.043*	
H25B	0.5295	0.6058	0.8285	0.043*	
C26	0.60791 (11)	0.5575 (2)	0.81072 (12)	0.0376 (6)	
H26A	0.6208	0.5684	0.8465	0.045*	
H26B	0.6379	0.5767	0.7869	0.045*	
C27	0.59455 (11)	0.44823 (19)	0.80301 (11)	0.0328 (6)	
H27A	0.5887	0.4335	0.7661	0.039*	
H27B	0.5609	0.4300	0.8224	0.039*	
C28	0.64732 (12)	0.3929 (2)	0.82466 (11)	0.0379 (6)	
H28B	0.6787	0.4027	0.8013	0.057*	
H28C	0.6391	0.3243	0.8270	0.057*	
H28A	0.6567	0.4175	0.8590	0.057*	
C29	0.64066 (10)	0.80479 (17)	0.74812 (14)	0.0385 (5)	
H29A	0.6808	0.8010	0.7409	0.046*	
H29B	0.6259	0.7387	0.7467	0.046*	
C30	0.50488 (16)	0.79714 (19)	0.89170 (10)	0.0404 (6)	
H30A	0.5013	0.8124	0.9288	0.049*	
H30B	0.4999	0.7272	0.8874	0.049*	
C31	0.49573 (17)	0.3828 (2)	0.52153 (11)	0.0460 (7)	
H31	0.4798	0.4157	0.4907	0.055*	
C32	0.73122 (15)	0.2694 (3)	0.01260 (14)	0.0508 (9)	
H32	0.7112	0.2278	0.0379	0.061*	
Cl1	0.48281 (3)	0.45236 (5)	0.57806 (3)	0.04247 (18)	
Cl2	0.46515 (3)	0.26789 (6)	0.52866 (3)	0.04539 (18)	
Cl3	0.56833 (3)	0.36925 (5)	0.51371 (3)	0.03910 (16)	
Cl4	0.68348 (3)	0.31971 (5)	−0.03167 (3)	0.04333 (18)	
Cl5	0.78106 (3)	0.20027 (5)	−0.02173 (3)	0.03840 (18)	
Cl6	0.76660 (3)	0.36324 (5)	0.04606 (3)	0.04280 (18)	
O1	0.57497 (8)	0.70222 (14)	0.67742 (8)	0.0400 (5)	
O2	0.55697 (8)	0.72678 (14)	0.79425 (8)	0.0401 (5)	
O3	0.7506 (3)	0.8728 (5)	0.6931 (3)	0.0465 (17)	0.30
H3A	0.7546	0.8515	0.6619	0.056*	0.30
H3C	0.7785	0.8548	0.7118	0.056*	0.30
O4	0.78185 (17)	0.7656 (3)	0.7477 (3)	0.0369 (9)	0.40
H4B	0.7729	0.7753	0.7796	0.044*	0.40
H4C	0.7675	0.7121	0.7372	0.044*	0.40
O5	0.4793 (3)	0.9277 (5)	0.9875 (3)	0.0464 (17)	0.30
H5B	0.5040	0.9180	1.0112	0.056*	0.30
H5C	0.4800	0.9873	0.9782	0.056*	0.30

### Atomic displacement parameters (Å^2^)

**Table:**

	*U*^11^	*U*^22^	*U*^33^	*U*^12^	*U*^13^	*U*^23^
C1	0.0446 (19)	0.0317 (16)	0.0495 (19)	−0.0136 (14)	0.0014 (14)	0.0140 (14)
C2	0.0419 (17)	0.0379 (16)	0.0416 (16)	−0.0079 (13)	0.0082 (13)	0.0112 (13)
C3	0.0405 (15)	0.0396 (15)	0.0358 (14)	−0.0037 (12)	0.0043 (12)	0.0091 (12)
C4	0.0529 (18)	0.0374 (15)	0.0359 (14)	−0.0077 (13)	0.0018 (13)	0.0115 (12)
C5	0.0389 (15)	0.0347 (14)	0.0260 (12)	−0.0067 (11)	0.0045 (11)	0.0022 (11)
C6	0.0319 (16)	0.0366 (16)	0.0425 (15)	0.0040 (12)	0.0091 (12)	−0.0020 (12)
C7	0.0435 (16)	0.0383 (15)	0.0342 (13)	0.0056 (12)	0.0009 (12)	0.0046 (12)
C8	0.0322 (14)	0.0398 (16)	0.0458 (16)	−0.0118 (12)	0.0055 (12)	0.0111 (13)
C9	0.0360 (15)	0.0355 (14)	0.0357 (13)	−0.0113 (10)	−0.0072 (10)	−0.0194 (11)
C10	0.0289 (14)	0.0411 (16)	0.0469 (16)	−0.0106 (12)	−0.0043 (12)	0.0161 (13)
C11	0.0383 (15)	0.0353 (14)	0.0366 (14)	0.0014 (12)	−0.0156 (12)	0.0012 (11)
C12	0.0314 (14)	0.0413 (16)	0.0477 (16)	−0.0030 (12)	0.0106 (12)	−0.0180 (13)
C13	0.0408 (15)	0.0374 (14)	0.0335 (13)	0.0079 (12)	0.0197 (12)	0.0100 (11)
C14	0.0299 (14)	0.0458 (16)	0.0356 (14)	−0.0042 (12)	0.0144 (12)	−0.0101 (12)
C15	0.0286 (15)	0.0514 (19)	0.0358 (16)	−0.0057 (14)	−0.0025 (12)	−0.0070 (14)
C16	0.0253 (14)	0.0437 (17)	0.0454 (17)	0.0022 (12)	−0.0003 (12)	−0.0089 (13)
C17	0.0395 (15)	0.0323 (14)	0.0353 (13)	−0.0079 (12)	−0.0132 (12)	0.0089 (11)
C18	0.0376 (16)	0.0488 (17)	0.0413 (15)	−0.0044 (13)	−0.0135 (13)	−0.0021 (13)
C19	0.0297 (14)	0.0364 (14)	0.0484 (16)	0.0098 (11)	−0.0061 (12)	−0.0129 (13)
C20	0.0357 (15)	0.0331 (14)	0.0345 (14)	0.0004 (12)	−0.0026 (12)	0.0082 (12)
C21	0.0351 (14)	0.0360 (15)	0.0339 (13)	−0.0060 (11)	−0.0089 (11)	0.0109 (11)
C22	0.0445 (17)	0.0413 (16)	0.0462 (16)	0.0042 (13)	0.0126 (14)	0.0092 (14)
C23	0.0322 (14)	0.0422 (16)	0.0428 (15)	−0.0106 (12)	0.0004 (12)	0.0092 (13)
C24	0.0405 (15)	0.0383 (15)	0.0373 (15)	0.0007 (12)	0.0051 (12)	0.0063 (12)
C25	0.0376 (15)	0.0336 (14)	0.0367 (14)	0.0063 (11)	0.0143 (12)	0.0080 (12)
C26	0.0305 (14)	0.0385 (15)	0.0438 (15)	−0.0084 (11)	−0.0140 (12)	0.0158 (12)
C27	0.0261 (13)	0.0320 (13)	0.0402 (14)	0.0011 (11)	0.0158 (11)	−0.0071 (11)
C28	0.0446 (16)	0.0305 (14)	0.0387 (14)	−0.0132 (12)	−0.0178 (12)	0.0062 (11)
C29	0.0395 (13)	0.0375 (13)	0.0385 (12)	−0.0099 (10)	0.0062 (17)	−0.0045 (16)
C30	0.0419 (16)	0.0379 (13)	0.0416 (13)	−0.0079 (16)	0.0082 (15)	0.0112 (10)
C31	0.0461 (17)	0.0377 (13)	0.0542 (15)	0.0044 (17)	−0.0183 (18)	−0.0080 (12)
C32	0.053 (2)	0.0443 (19)	0.0553 (19)	0.0173 (15)	−0.0097 (15)	−0.0163 (15)
Cl1	0.0439 (4)	0.0405 (4)	0.0431 (4)	−0.0083 (3)	0.0139 (3)	0.0118 (3)
Cl2	0.0457 (4)	0.0454 (4)	0.0451 (4)	0.0024 (3)	0.0075 (3)	0.0044 (3)
Cl3	0.0469 (4)	0.0377 (3)	0.0327 (3)	0.0113 (3)	0.0002 (3)	0.0123 (3)
Cl4	0.0435 (4)	0.0400 (4)	0.0464 (4)	0.0117 (3)	0.0128 (3)	−0.0054 (3)
Cl5	0.0459 (4)	0.0294 (3)	0.0399 (4)	0.0140 (3)	0.0162 (3)	0.0174 (3)
Cl6	0.0451 (4)	0.0454 (4)	0.0380 (3)	0.0105 (3)	0.0132 (3)	−0.0117 (3)
O1	0.0363 (11)	0.0351 (11)	0.0487 (12)	−0.0017 (8)	−0.0087 (9)	0.0058 (9)
O2	0.0401 (11)	0.0316 (11)	0.0485 (12)	0.0001 (9)	0.0012 (9)	−0.0122 (9)
O3	0.040 (4)	0.052 (4)	0.048 (4)	−0.025 (3)	0.006 (3)	0.021 (3)
O4	0.044 (2)	0.032 (2)	0.035 (2)	0.0053 (17)	−0.010 (3)	−0.002 (3)
O5	0.049 (4)	0.048 (4)	0.043 (4)	−0.012 (3)	0.014 (3)	−0.014 (3)

### Geometric parameters (Å, °)

**Table:**

C1—C6	1.381 (4)	C19—H19	0.9300
C1—C2	1.410 (5)	C20—C30	1.527 (4)
C1—O1	1.419 (3)	C21—C23	1.515 (4)
C2—C3	1.395 (4)	C21—C22	1.529 (4)
C2—C30^i^	1.546 (4)	C21—C24	1.539 (4)
C3—C4	1.389 (4)	C22—H22A	0.9600
C3—H3	0.9300	C22—H22B	0.9600
C4—C5	1.398 (4)	C22—H22C	0.9600
C4—C7	1.601 (4)	C23—H23A	0.9600
C5—C6	1.394 (4)	C23—H23B	0.9600
C5—H5	0.9300	C23—H23C	0.9600
C6—C29	1.449 (4)	C24—H24A	0.9600
C7—C10	1.499 (4)	C24—H24B	0.9600
C7—C8	1.507 (4)	C24—H24C	0.9600
C7—C9	1.523 (4)	C25—O2	1.475 (3)
C8—H8A	0.9600	C25—C26	1.523 (4)
C8—H8B	0.9600	C25—H25A	0.9700
C8—H8C	0.9600	C25—H25B	0.9700
C9—H9A	0.9600	C26—C27	1.540 (4)
C9—H9B	0.9600	C26—H26A	0.9700
C9—H9C	0.9600	C26—H26B	0.9700
C10—H10B	0.9600	C27—C28	1.562 (4)
C10—H10C	0.9600	C27—H27A	0.9700
C10—H10A	0.9600	C27—H27B	0.9700
C11—O1	1.403 (4)	C28—H28B	0.9600
C11—C12	1.520 (4)	C28—H28C	0.9600
C11—H11A	0.9700	C28—H28A	0.9600
C11—H11B	0.9700	C29—H29A	0.9700
C12—C13	1.507 (4)	C29—H29B	0.9700
C12—H12A	0.9700	C30—C2^i^	1.546 (4)
C12—H12B	0.9700	C30—H30A	0.9700
C13—C14	1.531 (4)	C30—H30B	0.9700
C13—H13A	0.9700	C31—Cl2	1.740 (3)
C13—H13B	0.9700	C31—Cl3	1.742 (4)
C14—H14B	0.9600	C31—Cl1	1.750 (3)
C14—H14C	0.9600	C31—H31	0.9800
C14—H14A	0.9600	C32—Cl4	1.737 (3)
C15—C20	1.370 (4)	C32—Cl5	1.746 (3)
C15—O2	1.407 (4)	C32—Cl6	1.754 (3)
C15—C16	1.420 (4)	C32—H32	0.9800
C16—C17	1.357 (4)	O3—H3A	0.8500
C16—C29	1.473 (4)	O3—H3C	0.8499
C17—C18	1.433 (4)	O4—H4B	0.8499
C17—H17	0.9300	O4—H4C	0.8500
C18—C19	1.331 (4)	O5—H5B	0.8500
C18—C21	1.550 (4)	O5—H5C	0.8499
C19—C20	1.415 (4)		
			
C6—C1—C2	120.6 (3)	C15—C20—C19	118.3 (3)
C6—C1—O1	123.5 (3)	C15—C20—C30	123.0 (3)
C2—C1—O1	115.9 (3)	C19—C20—C30	117.8 (2)
C3—C2—C1	119.4 (3)	C23—C21—C22	101.1 (2)
C3—C2—C30^i^	116.7 (3)	C23—C21—C24	107.3 (2)
C1—C2—C30^i^	123.6 (3)	C22—C21—C24	111.9 (2)
C4—C3—C2	119.2 (3)	C23—C21—C18	110.6 (2)
C4—C3—H3	120.4	C22—C21—C18	110.4 (2)
C2—C3—H3	120.4	C24—C21—C18	114.8 (2)
C3—C4—C5	121.6 (3)	C21—C22—H22A	109.5
C3—C4—C7	116.6 (3)	C21—C22—H22B	109.5
C5—C4—C7	121.8 (3)	H22A—C22—H22B	109.5
C6—C5—C4	118.8 (3)	C21—C22—H22C	109.5
C6—C5—H5	120.6	H22A—C22—H22C	109.5
C4—C5—H5	120.6	H22B—C22—H22C	109.5
C1—C6—C5	120.3 (3)	C21—C23—H23A	109.5
C1—C6—C29	113.5 (3)	C21—C23—H23B	109.5
C5—C6—C29	125.0 (3)	H23A—C23—H23B	109.5
C10—C7—C8	106.4 (2)	C21—C23—H23C	109.5
C10—C7—C9	110.4 (2)	H23A—C23—H23C	109.5
C8—C7—C9	111.6 (2)	H23B—C23—H23C	109.5
C10—C7—C4	109.6 (2)	C21—C24—H24A	109.5
C8—C7—C4	107.5 (2)	C21—C24—H24B	109.5
C9—C7—C4	111.2 (2)	H24A—C24—H24B	109.5
C7—C8—H8A	109.5	C21—C24—H24C	109.5
C7—C8—H8B	109.5	H24A—C24—H24C	109.5
H8A—C8—H8B	109.5	H24B—C24—H24C	109.5
C7—C8—H8C	109.5	O2—C25—C26	123.9 (2)
H8A—C8—H8C	109.5	O2—C25—H25A	106.4
H8B—C8—H8C	109.5	C26—C25—H25A	106.4
C7—C9—H9A	109.5	O2—C25—H25B	106.4
C7—C9—H9B	109.5	C26—C25—H25B	106.4
H9A—C9—H9B	109.5	H25A—C25—H25B	106.4
C7—C9—H9C	109.5	C25—C26—C27	110.6 (2)
H9A—C9—H9C	109.5	C25—C26—H26A	109.5
H9B—C9—H9C	109.5	C27—C26—H26A	109.5
C7—C10—H10B	109.5	C25—C26—H26B	109.5
C7—C10—H10C	109.5	C27—C26—H26B	109.5
H10B—C10—H10C	109.5	H26A—C26—H26B	108.1
C7—C10—H10A	109.5	C26—C27—C28	105.1 (2)
H10B—C10—H10A	109.5	C26—C27—H27A	110.7
H10C—C10—H10A	109.5	C28—C27—H27A	110.7
O1—C11—C12	111.2 (2)	C26—C27—H27B	110.7
O1—C11—H11A	109.4	C28—C27—H27B	110.7
C12—C11—H11A	109.4	H27A—C27—H27B	108.8
O1—C11—H11B	109.4	C27—C28—H28B	109.5
C12—C11—H11B	109.4	C27—C28—H28C	109.5
H11A—C11—H11B	108.0	H28B—C28—H28C	109.5
C13—C12—C11	111.5 (2)	C27—C28—H28A	109.5
C13—C12—H12A	109.3	H28B—C28—H28A	109.5
C11—C12—H12A	109.3	H28C—C28—H28A	109.5
C13—C12—H12B	109.3	C6—C29—C16	117.7 (2)
C11—C12—H12B	109.3	C6—C29—H29A	107.9
H12A—C12—H12B	108.0	C16—C29—H29A	107.9
C12—C13—C14	104.4 (2)	C6—C29—H29B	107.9
C12—C13—H13A	110.9	C16—C29—H29B	107.9
C14—C13—H13A	110.9	H29A—C29—H29B	107.2
C12—C13—H13B	110.9	C20—C30—C2^i^	110.8 (2)
C14—C13—H13B	110.9	C20—C30—H30A	109.5
H13A—C13—H13B	108.9	C2^i^—C30—H30A	109.5
C13—C14—H14B	109.5	C20—C30—H30B	109.5
C13—C14—H14C	109.5	C2^i^—C30—H30B	109.5
H14B—C14—H14C	109.5	H30A—C30—H30B	108.1
C13—C14—H14A	109.5	Cl2—C31—Cl3	109.10 (17)
H14B—C14—H14A	109.5	Cl2—C31—Cl1	109.46 (19)
H14C—C14—H14A	109.5	Cl3—C31—Cl1	108.92 (18)
C20—C15—O2	120.9 (3)	Cl2—C31—H31	109.8
C20—C15—C16	122.2 (3)	Cl3—C31—H31	109.8
O2—C15—C16	116.4 (3)	Cl1—C31—H31	109.8
C17—C16—C15	117.4 (3)	Cl4—C32—Cl5	109.38 (19)
C17—C16—C29	132.6 (3)	Cl4—C32—Cl6	109.55 (19)
C15—C16—C29	109.4 (2)	Cl5—C32—Cl6	108.37 (19)
C16—C17—C18	121.1 (3)	Cl4—C32—H32	109.8
C16—C17—H17	119.4	Cl5—C32—H32	109.8
C18—C17—H17	119.4	Cl6—C32—H32	109.8
C19—C18—C17	120.0 (3)	C11—O1—C1	111.9 (2)
C19—C18—C21	121.9 (3)	C15—O2—C25	128.4 (2)
C17—C18—C21	117.9 (3)	H3A—O3—H3C	109.5
C18—C19—C20	120.9 (3)	H4B—O4—H4C	109.5
C18—C19—H19	119.6	H5B—O5—H5C	109.5
C20—C19—H19	119.6		
			
C6—C1—C2—C3	−0.8 (5)	C16—C17—C18—C19	−3.7 (4)
O1—C1—C2—C3	−179.4 (3)	C16—C17—C18—C21	−179.1 (3)
C6—C1—C2—C30^i^	−173.7 (3)	C17—C18—C19—C20	1.0 (4)
O1—C1—C2—C30^i^	7.8 (4)	C21—C18—C19—C20	176.1 (3)
C1—C2—C3—C4	0.7 (4)	O2—C15—C20—C19	−175.9 (3)
C30^i^—C2—C3—C4	174.0 (3)	C16—C15—C20—C19	−4.2 (5)
C2—C3—C4—C5	−0.1 (4)	O2—C15—C20—C30	−7.0 (5)
C2—C3—C4—C7	179.5 (3)	C16—C15—C20—C30	164.8 (3)
C3—C4—C5—C6	−0.4 (4)	C18—C19—C20—C15	2.9 (4)
C7—C4—C5—C6	−180.0 (2)	C18—C19—C20—C30	−166.7 (3)
C2—C1—C6—C5	0.3 (5)	C19—C18—C21—C23	129.6 (3)
O1—C1—C6—C5	178.8 (3)	C17—C18—C21—C23	−55.2 (3)
C2—C1—C6—C29	168.3 (3)	C19—C18—C21—C22	−119.4 (3)
O1—C1—C6—C29	−13.2 (4)	C17—C18—C21—C22	55.9 (3)
C4—C5—C6—C1	0.3 (4)	C19—C18—C21—C24	8.1 (4)
C4—C5—C6—C29	−166.2 (3)	C17—C18—C21—C24	−176.6 (2)
C3—C4—C7—C10	172.7 (3)	O2—C25—C26—C27	−167.8 (2)
C5—C4—C7—C10	−7.7 (4)	C25—C26—C27—C28	−170.0 (2)
C3—C4—C7—C8	−72.0 (3)	C1—C6—C29—C16	−123.3 (3)
C5—C4—C7—C8	107.5 (3)	C5—C6—C29—C16	44.0 (4)
C3—C4—C7—C9	50.4 (3)	C17—C16—C29—C6	−77.3 (4)
C5—C4—C7—C9	−130.1 (3)	C15—C16—C29—C6	92.4 (3)
O1—C11—C12—C13	−168.8 (2)	C15—C20—C30—C2^i^	−73.2 (4)
C11—C12—C13—C14	−173.9 (2)	C19—C20—C30—C2^i^	95.8 (3)
C20—C15—C16—C17	1.5 (5)	C12—C11—O1—C1	−173.8 (2)
O2—C15—C16—C17	173.6 (3)	C6—C1—O1—C11	−77.5 (4)
C20—C15—C16—C29	−169.9 (3)	C2—C1—O1—C11	101.1 (3)
O2—C15—C16—C29	2.1 (4)	C20—C15—O2—C25	−80.2 (4)
C15—C16—C17—C18	2.4 (4)	C16—C15—O2—C25	107.6 (3)
C29—C16—C17—C18	171.5 (3)	C26—C25—O2—C15	−45.2 (4)

Symmetry codes: (i) −*x*+1, *y*, −*z*+3/2.

### Hydrogen-bond geometry (Å, °)

**Table:**

*D*—H···*A*	*D*—H	H···*A*	*D*···*A*	*D*—H···*A*
O3—H3C···O4	0.85	1.52	2.149 (8)	127
O5—H5C···O5^ii^	0.85	1.74	2.299 (13)	121

Symmetry codes: (ii) −*x*+1, −*y*+2, −*z*+2.
